# Emission and Accumulation of Monoterpene and the Key Terpene Synthase (TPS) Associated with Monoterpene Biosynthesis in *Osmanthus fragrans* Lour

**DOI:** 10.3389/fpls.2015.01232

**Published:** 2016-01-12

**Authors:** Xiangling Zeng, Cai Liu, Riru Zheng, Xuan Cai, Jing Luo, Jingjing Zou, Caiyun Wang

**Affiliations:** ^1^Key Laboratory for Biology of Horticultural Plants, Ministry of Education, College of Horticulture and Forestry Sciences, Huazhong Agricultural UniversityWuhan, China; ^2^School of Nuclear Technology and Chemistry and Biology, Hubei University of Science and TechnologyXianning, China

**Keywords:** *Osmanthus fragrans*, MEP pathway, terpene synthase, monoterpenes, glycosylation

## Abstract

*Osmanthus fragrans* is an ornamental and economically important plant known for its magnificent aroma, and the most important aroma-active compounds in flowers are monoterpenes, mainly β-ocimene, linalool and linalool derivatives. To understand the molecular mechanism of monoterpene production, we analyzed the emission and accumulation patterns of these compounds and the transcript levels of the genes involved in their biosynthesis in two *O. fragrans* cultivars during flowering stages. The results showed that both emission and accumulation of monoterpenes varied with flower development and glycosylation had an important impact on floral linalool emission during this process. Gene expression demonstrated that the transcript levels of terpene synthase (*TPS*) genes probably played a key role in monoterpene production, compared to the genes in the MEP pathway. Phylogenetic analysis showed that *Of*TPS1 and *Of*TPS2 belonged to a TPS-g subfamily, and *Of*TPS3 and *Of*TPS4 clustered into a TPS-b subfamily. Their transient and stable expression in tobacco leaves suggested that *Of*TPS1 and *Of*TPS2 exclusively produced β-linalool, and *trans*-β-ocimene was the sole product from *Of*TPS3, while *Of*TPS4, a predictive sesquiterpene synthase, produced α-farnesene. These results indicate that *OfTPS1*, *OfTPS2*, and *OfTPS3* could account for the major floral monoterpenes, linalool and *trans*-β-ocimene, produced in *O. fragrans* flowers.

## Introduction

Sweet *osmanthus (Osmanthus fragrans* Lour.), belonging to the Oleaceae family, is a well-known ornamental and economically important, aromatic woody plant, with the flower having a long history in China. Horticultural cultivars have spread throughout Thailand, India, and the Caucasus region (Baldermann et al., 2010). Because of its extremely powerful and unique aroma, flowers and the essential oils of *O. fragrans* are in high demand for the production of expensive perfumes, flavorings and cosmetics (Wang et al., 2009; Cai et al., 2014). The fresh flowers are very rich in floral volatiles, including terpenoids, aromatic compounds, C6 compounds and esters. The qualitative and quantitative variability of these compounds in flowers usually depends on the cultivar and developmental stage (Li et al., 2008; Cao et al., 2009; Sun et al., 2012; Xin et al., 2013). The terpenoids, including β-ionone, β-ocimene, β-linalool, and linalool derivatives, have been detected as dominant components of fresh flower volatiles and essential oils (Wang et al., 2009; Sun et al., 2012; Xin et al., 2013), and are important in the aroma formation of *O. fragrans* (Cai et al., 2014). Of the terpenes, β-ionone is ubiquitous in *O. fragrans* flowers and its biosynthesis has been reported at the molecular level (Baldermann et al., 2010; Han et al., 2014). However, the molecular mechanism for the formation of monoterpenes such as β-ocimene and linalool in *O. fragrans* is not clear.

In plants, monoterpenes are mainly synthesized through the plastidial methylerythritol 4-phosphate (MEP) pathway, providing terpene precursors isopentenyl diphosphate (IPP) and its allylic isomer, dimethylallyl diphosphate (DMAPP; Dudareva et al., 2013). Quantitative variation in monoterpene production can be controlled by substrate flux through the MEP pathway (Munoz-Bertomeu et al., 2006; Battilana et al., 2011; Külheim et al., 2011). The first step in the MEP pathway is the condensation of pyruvate and D-glyceraldehyde 3-phosphate (G3P) to 1-deoxy-D-xylulose 5-phosphate (DXP; Dudareva et al., 2013). The first enzyme, DXP synthase (DXS), has been considered the rate-limiting enzyme for the MEP pathway flux, because of the close correlation between the gene expression and the content of plastid isoprenoids such as monoterpenes and carotenoids (Estévez et al., 2001; Xie et al., 2008; Battilana et al., 2009). The second and sixth enzyme in the pathway, DXP reductoisomerase (DXR) and 4-hydroxy-3-methylbut-2-en-1-yl diphosphate synthase (HDS), are also potential regulatory control points (Mahmoud and Croteau, 2001; Carretero-Paulet et al., 2006; Külheim et al., 2011). However, the rate-limiting role of each of these enzymes in controlling the pathway flux appears to vary among plants (Cordoba et al., 2009).

Under the catalytic action of geranyl pyrophosphate synthase (GPPS), IPP, and DMAPP are condensed head-to-tail to produce geranyl diphosphate (GPP), the monoterpene substrate (Dudareva et al., 2013). Catalysis of this linear precursor, GPP, to a broad variety of monoterpenes is by the terpene synthase (TPS) family (Degenhardt et al., 2009). TPS enzymes from different plant species have distinct phylogenetic relationships and have been classified into seven subfamilies, designated *TPS*-a to *TPS*-g (Chen et al., 2011). Despite intriguing differences between the subfamilies, there are three conserved motifs: an arginine-rich N-terminal RR(x8)W motif required for cyclization of the GPP substrate; an aspartate-rich DDxxD motif that interacts with divalent metal (usually Mg^2+^ or Mn^2+^) ions involved in positioning the substrate for catalysis, and (N, D)Dxx(S,T)xxxE, required for second metal ion binding (Degenhardt et al., 2009). The RR(x8)W motif is involved in producing cyclic monoterpenes, and is absent in TPSs that produce acyclic products (Chen et al., 2011). Many TPSs have the ability to produce multiple terpenes from a single prenyl diphosphate substrate *in vitro* (Martin et al., 2010; Green et al., 2012; Nieuwenhuizen et al., 2013) or *in vivo* (Davidovich-Rikanati et al., 2008; Green et al., 2012). For example, the occasional complex terpene blend has been found in *Arabidopsis thaliana* and *Medicago truncatula*, often produced only by a limited number of multiproduct TPS enzymes (Tholl et al., 2005; Garms et al., 2010). To date, TPSs have been identified and characterized in many plants, including *Antirrhinum majus* (Dudareva et al., 2003; Nagegowda et al., 2008), *Actinidia* species (Nieuwenhuizen et al., 2009; Green et al., 2012) and *Vitis vinifera* (Martin et al., 2010). Despite monoterpenes making a significant contribution to the floral aroma and being rich in *O. fragrans* flowers, little is known about the *TPS* genes responsible for production of the major monoterpenes.

The biosynthetic monoterpenes are able to undergo complex processes of storage and conversion, which lead to the inconsistency between monoterpene release and gene transcript level (Chen et al., 2010; Green et al., 2012). Glycosides are a potential source of aroma and flavor compounds, and play important roles in controlling the release of the floral volatiles in flowers and fruit (Schwab et al., 2015). The glycoside volatiles are odorless and could release free aroma volatiles under the hydrolysis of β-glucosidase (Yauk et al., 2014; Schwab et al., 2015). They are highly valued in the flavor industry for enhancing the flavor and quality of grape wine and tea, and modifying the overall aroma during maturation, storage and processing in fruit (Birtić et al., 2009; Garcia et al., 2013; Yauk et al., 2014; Ohgami et al., 2015). In flowers, the organoleptic aroma contributed by free volatiles and the content of essential oil composed of non-glycoside volatiles accumulating in fresh flowers have received more attention compared to the few studies on the volatile glycoside (Picone et al., 2004; Green et al., 2012). It has been reported that glycosidically bound volatiles are more abundant than the free forms and are potentially a major source of aroma in flowers (Aurore et al., 2011; Wen et al., 2014; Yauk et al., 2014). Yang et al. (2005) found that β-D-glucosidase hydrolysis in fresh *O. fragrans* flowers enhanced the mass fractions of monoterpene volatiles. However, further research is needed on the glycoside monoterpenes in *O. fragrans*. Moreover, release of the bound volatile aglycones is dependent on flower development (Reuveni et al., 1999). Therefore, the connection between different forms of volatiles is fundamental for understanding the molecular mechanism of monoterpene biosynthesis.

Here, we give a detailed analysis of the emission and accumulation of floral monoterpenes in two *O. fragrans* cultivars during flowering. A total of 18 genes, including 13 MEP pathway genes from *DXS* to *IDI*, one *GPPS* and four *TPS* genes, were obtained by transcriptome sequencing and their expression levels were analyzed by real-time qPCR. Intriguing differences were found in the transcript levels of four *TPS*s. Furthermore, functional characterization of TPSs and their involvement in major monoterpene production in *O. fragrans* flowers are described. This work provides a better understanding of the molecular mechanism of monoterpene biosynthesis, and will also help in the biotechnological enhancement and modification of aroma in *O. fragrans*.

## Materials and Methods

### Plant Materials

The ‘Liuye Jingui’ (abbreviated as ‘Liuye’) and ‘Gecheng Dangui’ (abbreviated as ‘Gecheng’) cultivars of *O. fragrans* were grown in the campus nursery of Huazhong Agriculture University in Wuhan, China. Flower opening in *O. fragrans* was divided into four stages (Zou et al., 2014): tight bud stage (S1); initial flowering stage (S2); full flowering stage (S3); and late full flowering stage (S4). Flowers were harvested at about 10 a.m. in October 2013. Each sample at each flowering stages was separated into two parts. One was directly used for headspace volatile analysis, and the other was immediately frozen in liquid nitrogen for volatile solvent and glycoside extraction, and RNA extraction.

### SPME Collection and Solvent Extraction

The released floral volatiles were collected by solid-phase microextraction (SPME; Cai et al., 2014). In triplicate, 2 g fresh flowers were placed into a 20 ml capped SPME vial and incubated at 25 ± 2°C for 30 min. SPME fiber (50/30 μm DVB/CAR/PDMS on a 2 cm Stable Flex fiber, Supelco Inc. Bellefonte, PA, USA) was then exposed to the headspace of the capped vial for 30 min. The fiber was injected manually and desorbed in the injection port of the gas chromatograph (GC) with helium as the carrier gas. The fiber was desorbed for 5 min at 250°C in splitless mode. Before each set of samples was assayed, the fiber was conditioned for 1 h at 250°C in the injection port of the GC-MS and a fiber blank recorded.

The accumulated free floral volatiles were collected by solvent extraction (Green et al., 2012). In triplicate, 2 g frozen flowers, harvested at the equivalent time points to the SPME sampling, were ground to a fine powder in liquid nitrogen, transferred to a 50 ml centrifuge tube and extracted twice with 10 ml pentane/Et2O (1:1 v/v) for 30 min with gentle shaking. The two extractions were combined and stored overnight at -20°C. The following day, the upper solvent layer was carefully separated from the lower frozen water layer and reduced to 2 ml under a gentle stream of nitrogen. The concentrated extract, with 47.3 ng/μl cyclohexanone added as internal standard, was passed through a column of anhydrous MgSO_4_ to remove any remaining water, and then injected into the GC.

### Glycoside Extraction

Glycoside analysis was carried out according to Green et al. (2012) with minor modifications. In triplicate 2 g frozen flowers, harvested at the equivalent time points to the headspace sampling, were ground to a fine power in liquid nitrogen, transferred to a 50 ml centrifuge tube and resuspended in 30 ml ddH_2_O. The sample was centrifuged at 8,000 *g* for 15 min at 4°C and the supernatant run on a 15 mm × 25 mm i.d. Amberlite XAD-2 column (Supelco, Bellefonte, PA, USA) according to the manufacturer’s instructions, at the rate of 3 ml/min. Soluble sugars and acids were removed with 40 ml water and free volatiles by the addition of 40 ml pentane/Et2O (1:1 v/v). Bound glycosides were eluted with 20 ml methanol and evaporated to dryness in a rotary evaporator. The resulting glycoside pellet was resuspended in 2 ml deglycosylation buffer (200 mM Na_2_HPO_4_, 220 mM citric acid, pH 5.0) and re-extracted three times with 1 ml pentane/Et2O (1:1 v/v) to remove any remaining free compounds.

Enzymatic hydrolysis was carried out using β-glucosidase (6 u/mg; Sigma–Aldrich, Co, LLC, USA), dissolved in deglycosylation buffer, at a concentration of 10 mg/ml. The hydrolysis sample was overlaid with 1 ml Et2O and incubated at 40°C for 36 h. Following incubation, the sample was extracted a further three times with 1 ml Et2O. Prior to GC-MS analysis, the pooled extracts, with the addition of 47.3 ng/μl cyclohexanone as internal standard, were passed through a column of anhydrous MgSO_4_ and reduced to 0.5 ml under a gentle stream of nitrogen.

### GC-MS Analysis

The samples of the SPME collected, solvent extracted (1 μl) and glycoside volatiles (1 μl) were separated on a 30 m × 0.25 mm × 0.25 μm HP-5 capillary column (Thermo Scientific, Bellefonte, PA, USA). The system was a TRACE GC Ultra GC coupled to a DSQ II mass spectrometer (Thermo Fisher Scientific, Waltham, MA, USA). The GC-MS was performed according to Cai et al. (2014). The GC oven ramp for SPME collected volatiles was at 40°C for 3 min, 3°C/min to 73°C and held for 3 min, 5°C/min to 220°C and held for 1 min. The GC oven ramp for the solvent extracted and glycoside volatiles was at 40°C for 3 min, 3°C/min to 73°C and held for 3 min, 5°C/min to 240°C and held for 10 min. The flow rate of the helium (99.999%) carrier gas was 1.2 ml/min. The transfer line temperature was 280°C. For the mass detector, the ion source temperature was set at 230°C, with electronic impact (EI) mode at 70 eV over the mass range m/z 40–450 amu. A C8–C40 alkane standard solution (Sigma–Aldrich, Co., LLC., USA) was analyzed regularly to provide references for calculation of retention time (Kovats) indices (RIs) and to monitor system performance. Identification of the compounds was based on a comparison of their mass spectra and retention indices (RIs) with the authentic standards and published data, as well as standard mass spectra in the NIST05. Relative quantification of the target compounds for emission was by measuring peak areas, and for accumulation using the internal standard method.

### RNA Extraction and Real-Time PCR

Total RNA was isolated from 0.20 g frozen flowers using TRIzol reagent (CoWin Biotech Co., Ltd., Beijing, China), following the manufacturer’s instructions, and then treated with RQ1 DNase I (Promega, Madison, WI, USA) to remove genomic DNA. To synthesize first-strand cDNA, 3.50 μg total RNA was used with the RevertAid^TM^ First Strand cDNA Synthesis Kit (Fermentas, Thermo Fisher Scientific Inc., USA) according to the manufacturer’s instructions. The synthetic first-strand cDNAs were diluted 10-fold for gene expression analysis.

Gene expression was detected by qRT-PCR in both ‘Gecheng’ and ‘Liuye’ flowers at four flowering stages. The qRT-PCR was performed on an Applied Biosystems 7500 Fast Real-Time PCR platform with the SYBR^®^ Premix Ex Taq^TM^ II mix (Takara Biotechnology Co., Ltd., Dalian, China), according to the manufacturer’s instructions, and the results were analyzed using the Applied Biosystems 7500 software (Applied Biosystems Life Technologies). Three biological replicates were tested, and reactions carried out in triplicate. Relative transcript levels were calculated by the 2^-ΔΔCt^ method using β*-actin* as the endogenous control gene for data normalization. The primers for qRT-PCR analysis are listed in Supplemental Table S2.

### Isolation of *OfTPS* Genes

Based on the *TPS* unigene sequences from the transcriptome sequencing of *O. fragrans* flowers, the full-lengths of four *TPS* genes were obtained using the SMARTER^TM^ RACE method. The 5′ and 3′-RACE-Ready cDNAs were separately synthesized using the BD SMARTER^TM^ RACE cDNA Amplification Kit (Clontech, Mountain View, CA, USA). The amplified *OfTPS*s sequences were cloned into pEASY-T1 (TransGene Biotech CO., LTD, Beijing, China) and at least three independent clones were sequenced to check for PCR errors. The *OfTPS*s open reading frame (ORF) was predicted using the NCBI ORF Finder^1^. All primers used are listed in Supplemental Table S2.

### Multiple Sequence Alignment and Phylogenetic Analysis

The DNAMAN 6.0 software (Lynnon Biosoft, USA) was used for multiple sequence alignment, and the phylogenetic tree constructed using the default parameters of the MEGA 6.1 software. The MEGA employed Clustal W2 software to generate multiple alignments and construction of the phylogenetic tree was based on the neighbor-joining computational method with 1000 bootstrap replicates. The bioinformatics tools ChloroP^2^ and TargetP^3^ were used to predict the intracellular localization of *Of*TPS proteins.

### Transient and Stable Expression of *OfTPS* Genes in Tobacco

Full-length *OfTPS* ORFs were obtained from the pESAY-T1 vectors containing the target genes, using FastDigest enzymes (Fermentas, Thermo Fisher Scientific Inc., USA). *OfTPS1*, *OfTPS2*, and *OfTPS3* were digested with KpnI-XbaI, and *OfTPS4* with SmaI-XbaI. The restriction enzyme-generated inserts were cloned into the same restriction sites of the pCAMBIA 2300 binary vector to create pCAMBIA 2300::*OfTPSs* using T4 DNA ligase (Fermentas, Thermo Fisher Scientific Inc., USA). pCAMBIA 2300 contained the CaMV 35S promoter and nos-terminator. The pCAMBIA 2300::p19 was created by digesting the pGH-p19 vector using SmaI-XbaI to clone into the pCAMBIA 2300 vector, as described above. These plasmids were transformed in *Agrobacterium tumefaciens* strain EHA105 by electroporation.

Four to 6-week-old greenhouse-grown *Nicotiana benthamiana* seedlings were infiltrated with the *A. tumefaciens* strain EHA105, harboring the pCAMBIA 2300::*OfTPSs* and pCAMBIA 2300::p19, as described previously (Hellens et al., 2005). When freshly grown *Agrobacterium* cultures reached an OD_600__nm_ of 0.6–0.8, they were centrifuged and resuspended in infiltration media (10 mM MES, 10 mM MgCl_2_, 200 μM acetosyringone). The suspensions were adjusted to an OD_600__nm_ of between 1.0 and 2.0, and incubated without shaking at 28°C for 2 h. The target gene and viral suppressor p19 *Agrobacterium* cultures were mixed in a 1:1 ratio before injection into *N. benthamiana* leaves using a syringe. After 5 days, 2 g of the treated leaves were harvested and placed in a 20 ml SPME vial for volatile analysis (as above).

Leaf disks of *N. tabacum* were transformed by co-culture with *A. tumefaciens* strain EHA105 harboring the pCAMBIA 2300::*OfTPSs* binary vector. Three to five independent transformed lines were obtained. The transformed plants, obtained after selection with kanamycin, were confirmed by semi quantitative RT-PCR with 2x Es Taq MasterMix (CoWin Biotech Co., Ltd, Beijing, China) and GAPDH as the reference gene. 2 g leaves of the transformed plants were used for volatile analysis.

## Results

### Analysis of Monoterpene Emission and Accumulation in *O. fragrans* Flowers

**FIGURE 1:**
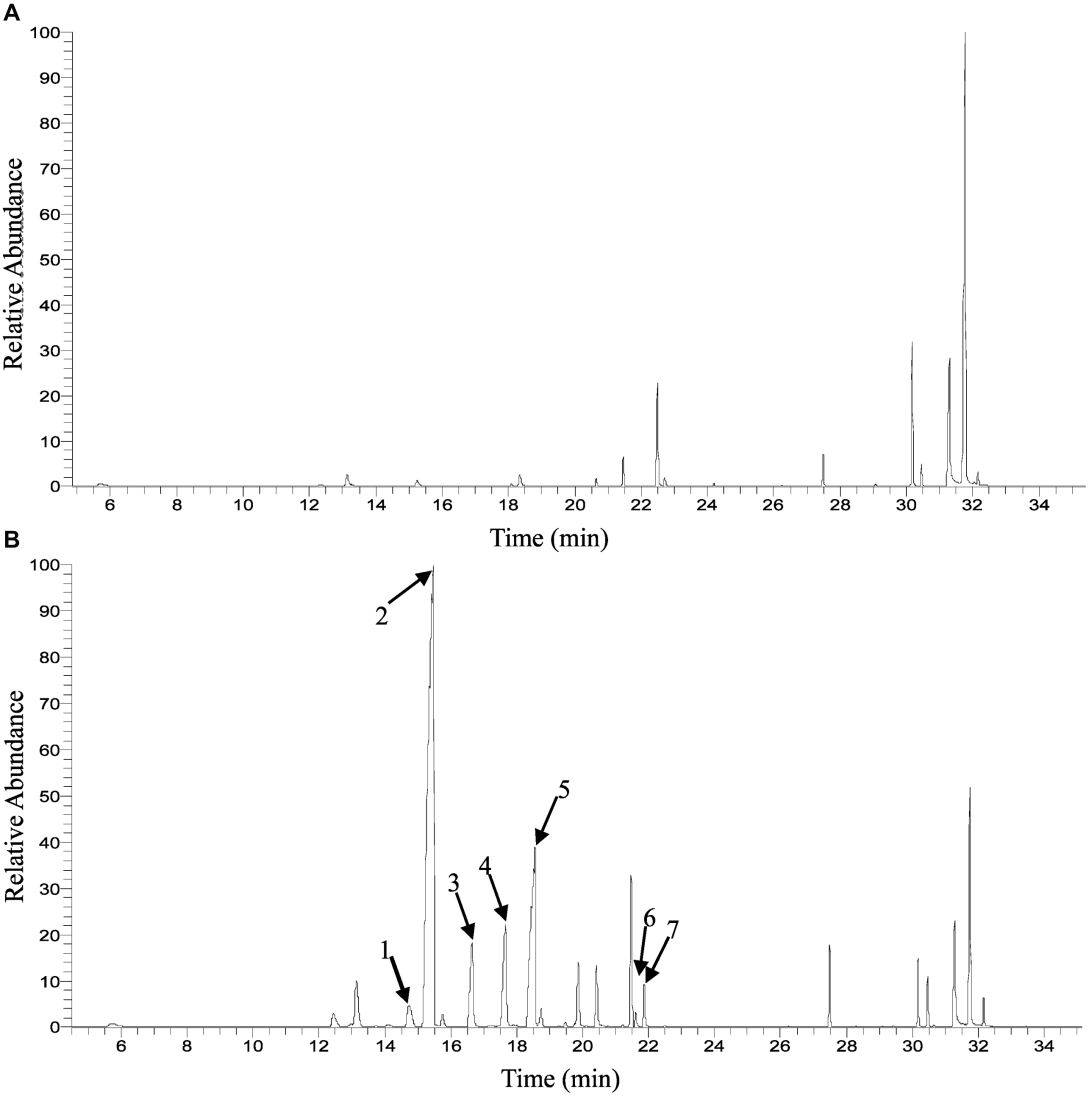
**Gas chromatograph (GC) trace of floral volatile emission in *Osmanthus fragrans* at the initial flowering stage (S2). (A)** Liuye, **(B)** Gecheng. (1) *cis*-β-ocimene; (2) *trans*-β-ocimene; (3) *cis*-Linalool oxide (furanoid); (4) *trans*-linalool oxide (furanoid); (5) β-linalool; (6) *cis*-linalool oxide (pyranoid); (7) *trans*-linalool oxide (pyranoid).

**FIGURE 2:**
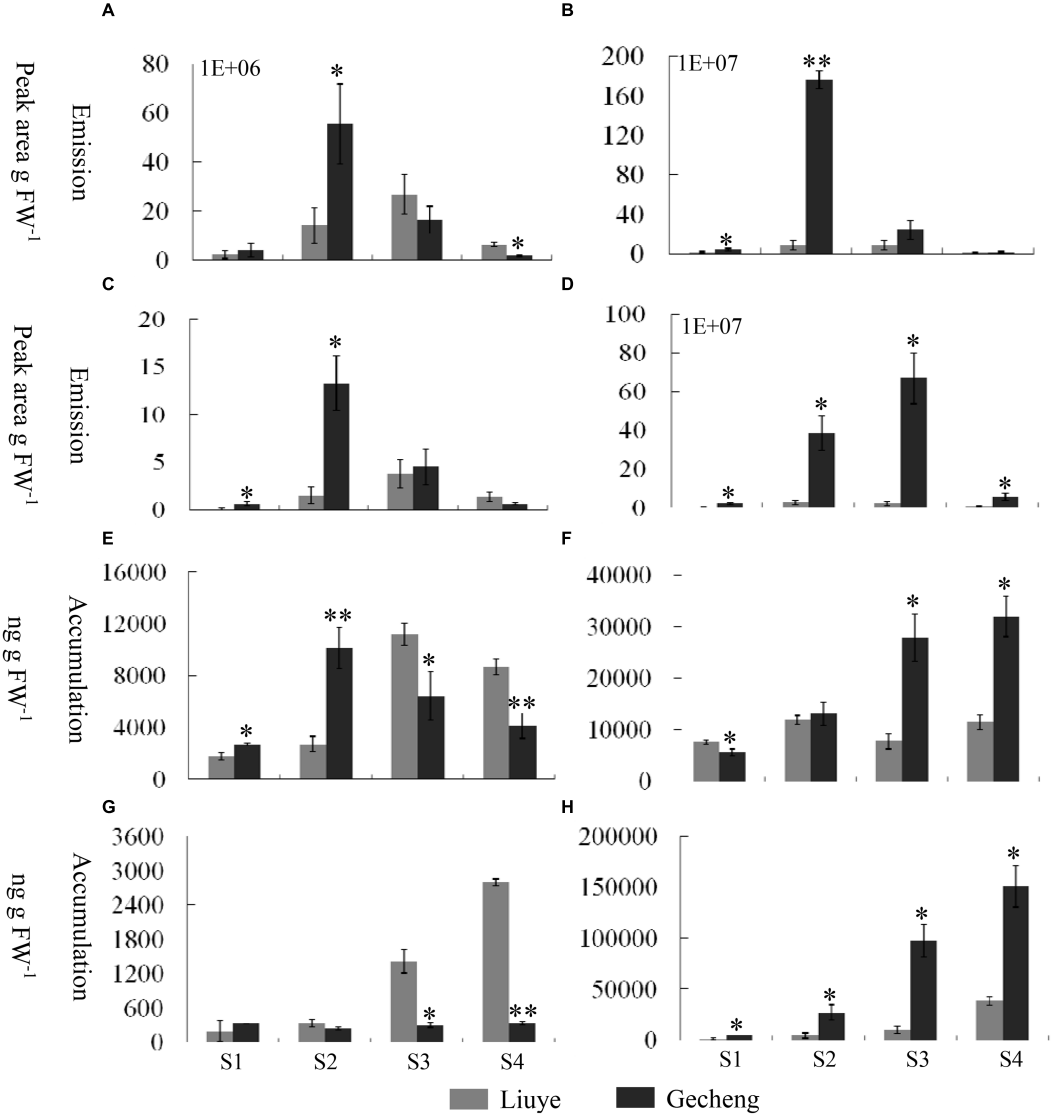
**Emission and accumulation of monoterpenes and their derivatives in *O. fragrans* flowers at four stages. (A–D)** Emission abundance of monoterpenes and their derivative volatiles: **(A)** *cis*-β-ocimene; **(B)** *trans*-β-ocimene; **(C)** linalool, and **(D)** total linalool derivatives. **(E,F)** Free accumulation of linalool and its derivatives: **(E)** linalool and **(F)** total linalool derivatives. **(G,H)** Glycoside accumulation of linalool and its derivatives: **(G)** linalool and **(H)** total linalool derivatives. The four flowering stages are: (S1) tight bud stage; (S2) initial flowering stage; (S3) full flowering stage, and (S4) late full flowering stage. Data are presented as mean ± SE (*n* = 3). The asterisks indicate significant differences between the values of ‘Liuye’ and ‘Gecheng’ at a given flower stage calculated by the Student’s *t*-test (^∗^*P* < 0.05, ^∗∗^*P* < 0.01).

**FIGURE 3:**
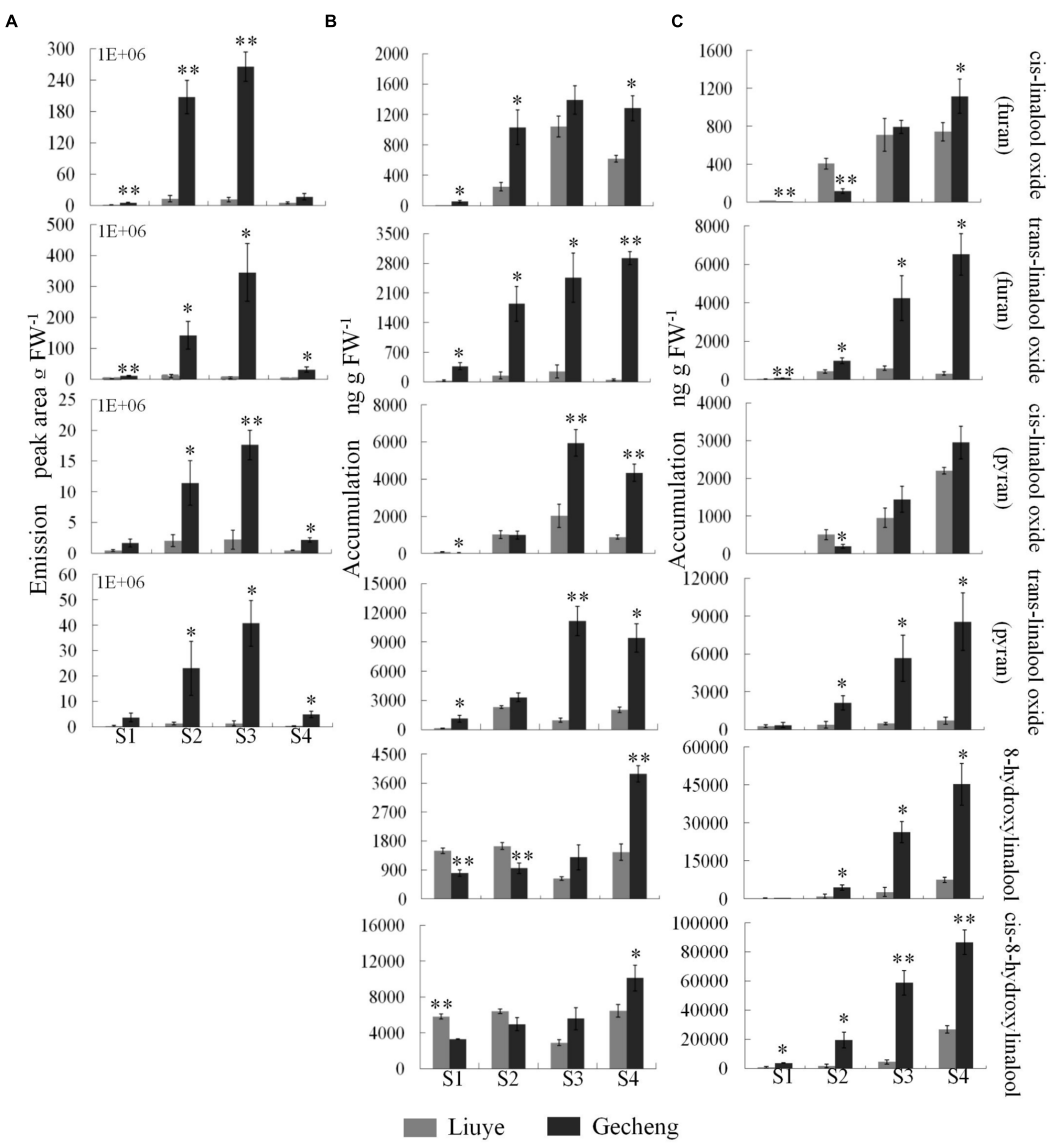
**Emission and accumulation of linalool derivatives in *O. fragrans* flowers at four stages. (A)** Emission of linalool derivatives. **(B)** Accumulation of free linalool derivatives. **(C)** Accumulation of glycosylated linalool derivatives. Flower stages are: (S1) tight bud stage; (S2) initial flowering stage; (S3) full flowering stage, and (S4) late full flowering stage. Data are presented as mean ± SE (*n* = 3). The asterisks indicate significant differences between the values of ‘Liuye’ and ‘Gecheng’ at a given flower stage calculated by the Student’s *t*-test (^∗^*P* < 0.05, ^∗∗^*P* < 0.01).

Solid-phase microextraction-GC-MS analysis identified a total of 33 volatile compounds in two cultivars of ‘Gecheng’ and ‘Liuye’ at the initial flowering stage (S2), assigned to monoterpene, norisoprenoid, aromatic and fatty acid-related compounds (Supplemental Table S1). Seventeen monoterpenes were found in the two cultivars, with β-ocimene, linalool and derivatives the dominant components in both cultivars. However, the content of monoterpenes differed in the two cultivars (**Figure 1**). The relative content of total monoterpenes was higher in ‘Gecheng’ (70%) than ‘Liuye’ (5%). In particular, *trans*-β-ocimene and linalool accounted for 44 and 23% of the total volatiles in ‘Gecheng’, but for only 1 and 2% in ‘Liuye’ (Supplemental Table S1). The emission of *cis*-β-ocimene, *trans*-β-ocimene, linalool and linalool derivatives in both ‘Gecheng’ and ‘Liuye’ flowers showed a similar pattern, increasing from S1 to S3 and decreasing at S4 (**Figures 2A–D** and **3A**). The peak of β-ocimene and linalool emission occurred at S2 in ‘Gecheng’ and at S3 in ‘Liuye,’ while the peak of the linalool derivatives in both cultivars was at S3. The β-ocimene and linalool emissions at S2 were much higher in ‘Gecheng’ than ‘Liuye.’ The emissions of all linalool derivatives were higher in ‘Gecheng’ throughout the flowering stages.

The accumulation of free monoterpenes in flowers was analyzed by solvent extraction, combining with GC-MS, collecting samples at the same stage as for SPME (**Figures 2E,F** and **3B**). Compared with the emitted monoterpenes, β-ocimene was barely detectable, but an abundance of linalool and derivatives accumulated in *O. fragrans* flowers. *Cis*-8-hydroxylinalool and 8-hydroxylinalool, the linalolol derivatives, were not detected as emitted floral volatiles but were found in solvent extracts. The accumulation pattern of linalool was consistent with the emission pattern in both cultivars, increasing from S1 to S3 and decreasing slightly at S4. The accumulation of linalool oxides continuously increased up to S3 and slightly decreased at S4, except for *trans*-linalool oxide(fur) in ‘Gecheng,’ which was still increasing at S4. The accumulation of *cis*-8-hydroxylinalool and 8-hydroxylinalool in ‘Gecheng’ gradually increased during the flowering stages, reaching the maximum at S4, while they basically remained steady in ‘Liuye,’ dropping slightly at S3. More linalool was accumulated in ‘Gecheng’ at S1 and S2, but in ‘Liuye’ at S3 and S4. The result was just the contrary to *cis*-8-hydroxylinalool and 8-hydroxylinalool. There was much more accumulation of linalool oxides in ‘Gecheng’ compared to ‘Liuye’ during flowering.

Floral glycosylated volatiles were extracted from *O. fragrans* flowers and analyzed by GC-MS after enzymatic hydrolysis (**Figures 2G,H** and **3C**; **Table 1**). Due to lack of hydoxylation in β-ocimene, only linalool and its derivatives were detected in glycosylated forms in *O. fragrans* flowers. In both cultivars, the glycosylated linalool and derivatives continuously increased during flowering, except for linalool in ‘Gecheng,’ which remained stable throughout the flowering stages. After S2, more glycosylated linalool accumulated in ‘Liuye’ than in ‘Gecheng.’ The glycosylated linalool derivatives as *trans*-linalool oxide (furan and pyran), *cis*-8-hydroxylinalool and 8-hydroxylinalool accumulated much more in ‘Gecheng’ than ‘Liuye’ throughout the flowering process. The percentage of glycosylation of linalool continued increasing in ‘Liuye’ from S1 to S4, but maintained a lower fraction in ‘Gecheng.’ Although the percentage of glycoside linalool oxides changed irregularly, total linalool derivatives in glycoside forms continued increasing in both cultivars from S1 to S4, due to the dominant components, *cis*-8-hydroxylinalool and 8-hydroxylinalool, were constantly increasing.

**Table 1:** Glycosylation of linalool and its derivatives in ‘Liuye’ and ‘Gecheng’ cultivars of *Osmanthus fragrans* flowers at four stages.

	Glycoside (% of total accumulation)							
	Liuye				Gecheng			
Compounds	S1	S2	S3	S4	S1	S2	S3	S4
β-linalool	10.03 ± 2.19	10.97 ± 0.15	11.22 ± 1.43	24.44 ± 1.22	10.96 ± 0.39	** 2.36 ± 0.24**	** 4.49 ± 1.05**	** 7.60 ± 1.54**
Total linalool derivatives	14.89 ± 3.16	27.19 ± 3.42	55.71 ± 11.74	76.88 ± 4.93	**43.69 ± 8.50**	**67.58 ± 12.70**	77.76 ± 7.56	82.52 ± 6.10
*Cis*-linalool oxide (furan)	66.44 ± 5.36	61.97 ± 4.38	40.56 ± 8.36	54.65 ± 5.56	**12.79 ± 2.70**	**10.29 ± 0.37**	36.33 ± 1.07	46.56 ± 5.80
*Trans*-linalool oxide (furan)	51.10 ± 12.30	73.80 ± 1.38	70.51 ± 9.60	86.62 ± 10.76	**18.39 ± 2.41**	**34.81 ± 0.37**	63.21 ± 12.35	69.03 ± 8.18
*Cis*-linalool oxide (pyran)	15.19 ± 2.63	33.06 ± 6.81	32.04 ± 5.03	71.45 ± 1.72	26.43 ± 7.34	**16.82 ± 2.84**	**19.50 ± 4.16**	**40.49 ± 4.73**
*Trans*-linalool oxide (pyran)	67.29 ± 15.68	13.70 ± 2.16	33.40 ± 2.70	25.89 ± 6.30	**24.10 ± 7.80**	**38.85 ± 8.91**	33.63 ± 9.82	47.56 ± 10.48
8-hydroxylinalool	13.14 ± 3.40	37.58 ± 11.50	81.03 ± 12.90	83.73 ± 5.82	**29.65 ± 6.13**	**82.49 ± 10.55**	95.33 ± 4.34	92.08 ± 6.48
*Cis*-8-hydroxylinalool	11.60 ± 1.58	21.06 ± 5.45	60.09 ± 14.33	80.53 ± 4.32	**52.29 ± 12.31**	**79.63 ± 13.24**	**91.32 ± 5.09**	89.54 ± 3.64

*Data are presented as mean ± SE (*n* = 3). The values in bold indicate significant differences to the ‘Liuye’ values at a given flower stage ( Student’s *t*-test *P* < 0.05)*.

### Expression Analysis of Genes Involved in Monoterpene Biosynthesis in *O. fragrans* Flowers

**FIGURE 4:**
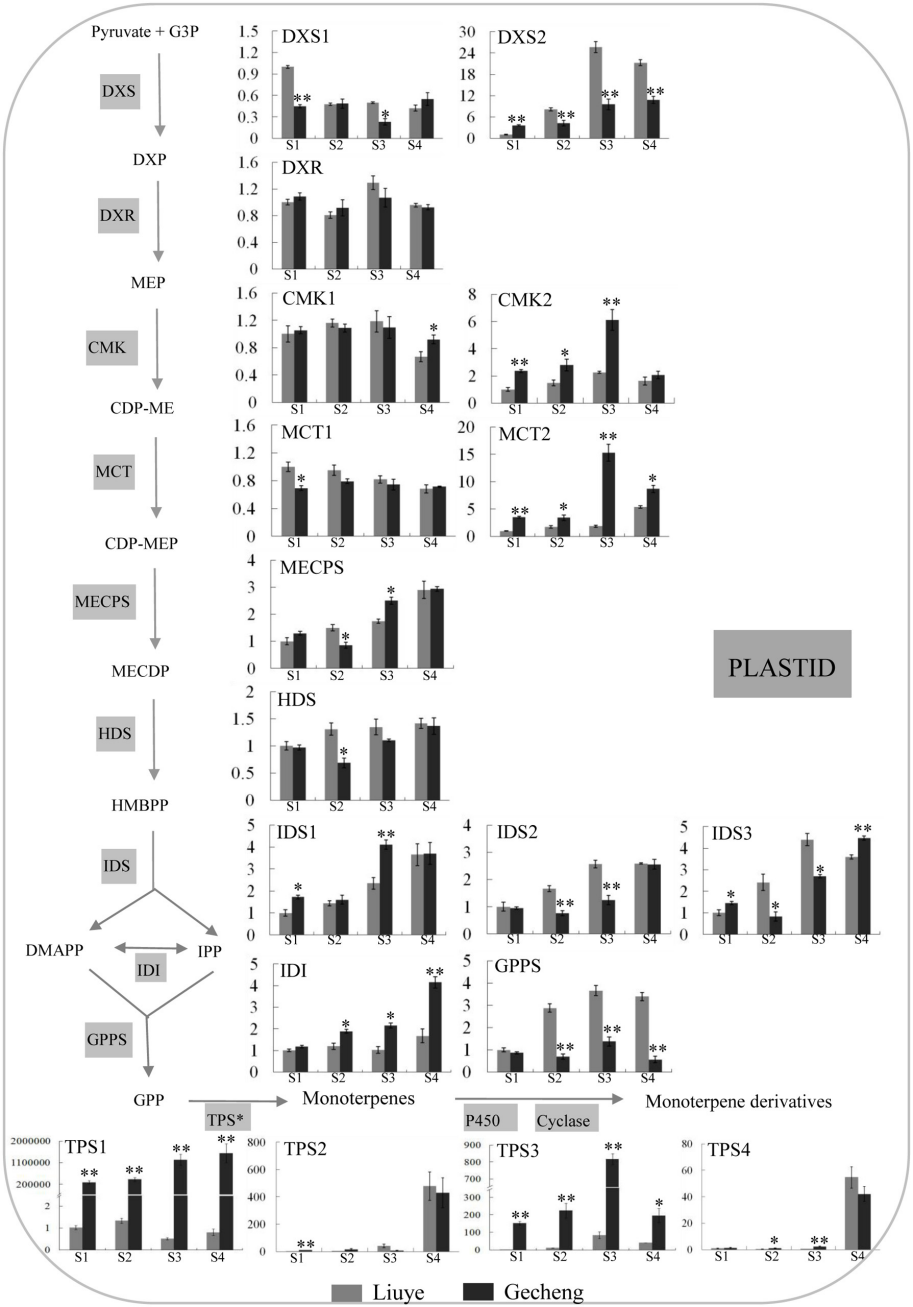
**Expression of genes involved in monoterpene biosynthesis in *O. fragrans* flowers at four stages**. Flower stages are: (S1) tight bud stage; (S2) initial flowering stage; (S3) full flowering stage, and (S4) late full flowering stage. The relative abundance was determined using a 2^-ΔΔCt^ method with β-actin as the reference gene. Results represent the mean ± SE of three technical repetitions and three biological replicates. The asterisks indicate significant differences between the values of ‘Liuye’ and ‘Gecheng’ at a given flower stage calculated by the Student’s *t*-test (^∗^*P* < 0.05, ^∗∗^*P* < 0.01). Abbreviations: G3P, glyceraldehyde 3-phosphate; DXP, 1-deoxy-D-xylulose 5-phosphate; MEP, methylerythritol phosphate; CDP-ME, 4-diphosphocytidyl-2-C-methyl-D-erythritol; CDP-MEP, CDP-ME 2-phosphate; MECPD, 2-C-methyl-D-erythritol 2,4-cyclodiphosphate; HMBPP, (E)-4-hydroxy-3-methylbut-2-en-1-yl diphosphate; IPP, isopentenyl pyrophosphate; DMAPP, dimethylallyl pyrophosphate; GPP, geranyl pyrophosphate; MVP, mevalonate 5-phosphate; FPP, farnesyl pyrophosphate; DXS, 1-deoxy-D-xylulose 5-phosphate synthase; DXR, 1-deoxy-D-xylulose 5-phosphate reductoisomerase; MCT, 2-C-methyl-D-erythritol 4-phosphate cytidylyltransferase; CMK, 4-(cytidine 5′-diphospho)-2-*C*-methyl-D-erythritol kinase; MECPS, 2-*C*-methyl-D-erythritol 2,4-cyclodiphosphate synthase; HDS, 4-hydroxy-3-methylbut-2-en-1-yl diphosphate synthase; IDS, isopentenyl diphosphate synthase; IDI, isopentenyl pyrophosphate isomerase; GPPS, geranyl pyrophosphate synthase; TPS, terpene synthase.

The MEP pathway produces IPP and DMAPP for production of monoterpenes (Munoz-Bertomeu et al., 2006) and TPS is the final enzyme converting the precursor GPP to kinds of monoterpenes (Degenhardt et al., 2009). The transcript levels of thirteen genes involved in the eight enzyme reaction stages of the MEP pathway, one *GPPS*, and four *TPS*s were analyzed by real-time qPCR (**Figure 4**).

In the MEP pathway, the expression levels of *DXR*, *CMK1*, and *MCT1* remained stable in the two cultivars throughout the flowering stages, and only subtle differences (<2 fold) were detected in *DXS1*, *MECPS*, *HDS*, *IDS1*, *IDS2*, and *IDS3* expression between the two cultivars. There was higher expression of *CMK2*, *MCT2*, and *IDI* in ‘Gecheng’ than ‘Liuye’ from S1 to S4, but the difference was no more than threefold between the two cultivars, except for *MCT2* at S3. *DXS2* expression was no more than threefold higher in ‘Liuye’ than ‘Gecheng’ from S2 to S4. These results showed that, during flowering, there were minor differences between the two cultivars in the expression of most genes in the MEP pathway. Expression of *GPPS* was the same in both cultivars at S1, but three–fivefold higher in ‘Liuye’ from S2 to S4. Regarding the *TPS* genes, there was no apparent difference between the two cultivars during flowering for *TPS2* and *TPS4* expression, which gradually increased from S1 to S3 followed by a dramatic increase at S4. However, it is worth noting that the expression of *TPS1* and *TPS3* was more than 100-fold higher in ‘Gecheng’ compared to ‘Liuye’ throughout flowering stage. *TPS1* expression consistently increased from S1 to S4, while *TPS3* had the highest level of transcript accumulation at S3 in the two cultivars. The differences in the levels of *TPS* gene expression between ‘Gecheng’ and ‘Liuye’ indicated their important contribution to monoterpene formation in *O. fragrans* flowers.

### Sequence Characterization of *TPS* Genes from *O. fragrans* Flowers

**FIGURE 5:**
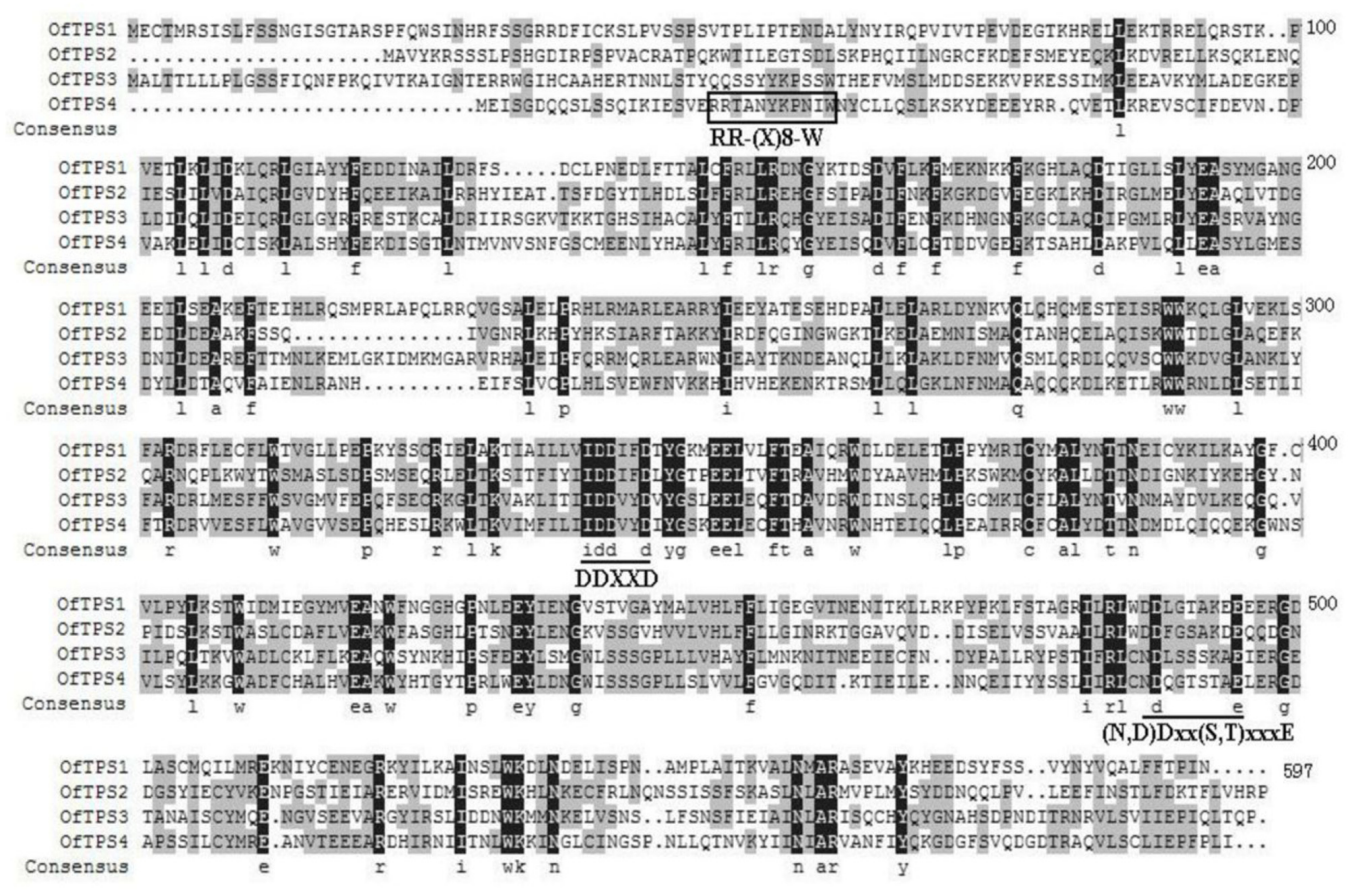
**Alignment of the amino acid sequences of *Of*TPS1, *Of*TPS2, *Of*TPS3, and *Of*TPS4**. Amino acids identical in all four proteins are shaded black and those identical in three proteins are shaded gray. The three highly conserved motifs are labeled RR-(x)_8_-W, DDxxD and (N,D)Dxx(S,T)xxxE, respectively.

**FIGURE 6:**
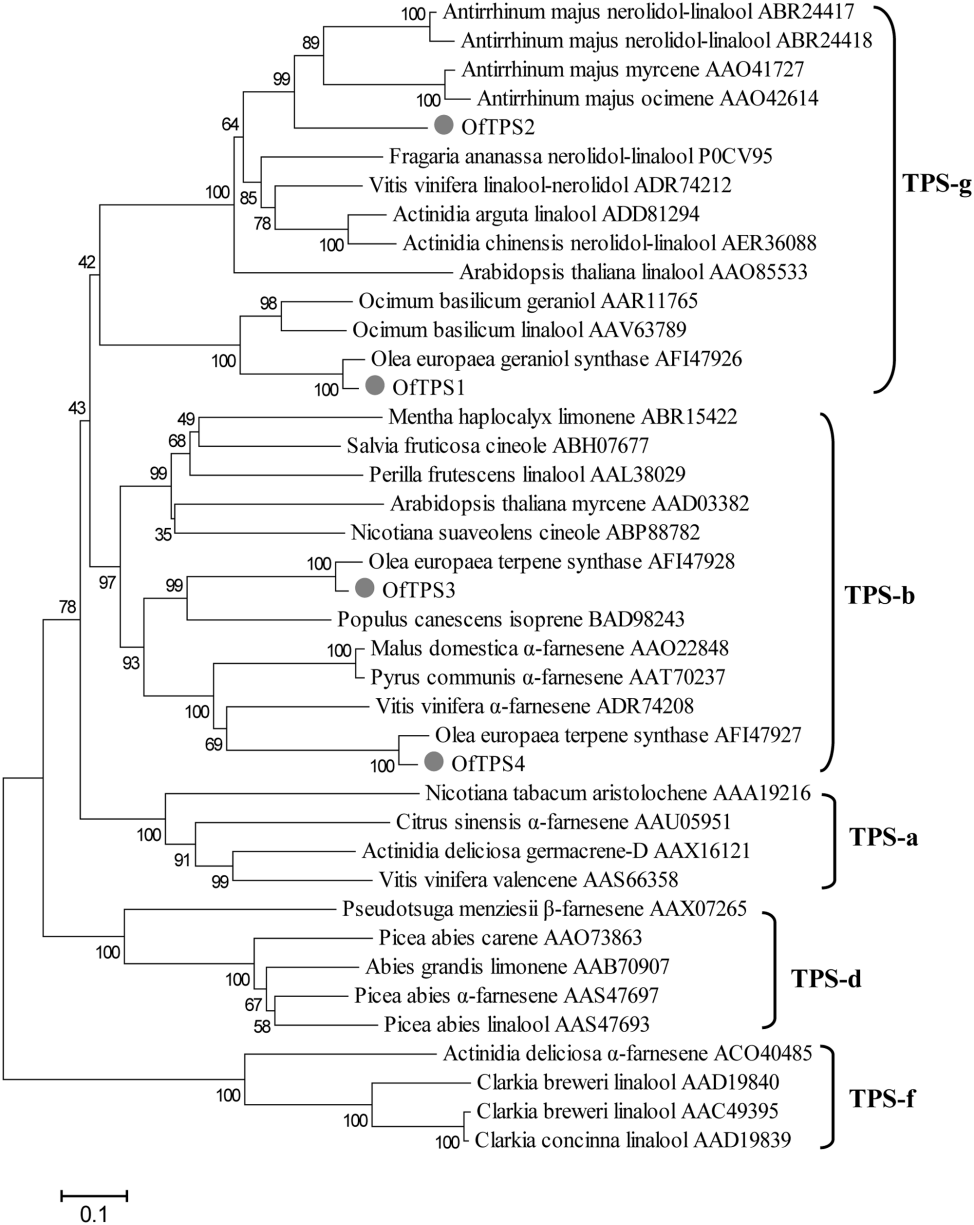
**Phylogenetic analysis of selected plant terpene synthases (TPSs) based on estimation of pair-wise distances at the amino acid level**. Clusters corresponding to five TPS subfamilies (TPS-a,TPS-b, TPS-d, TPS-g, and TPS-f) are apparent. The Clustal W 2.0 algorithm was used for the alignment. Trees were inferred with the neighbor-joining (NJ) method and *n* = 1000 replicates for bootstrapping.

The four full-length *TPS* genes were cloned using the RACE-PCR method. Their ORF sequences were 1746, 1668, 1776, and 1647 bp, respectively, designated *OfTPS1*, *OfTPS2*, *OfTPS3*, and *OfTPS4* (GeneBank no. KT591180 to KT591183). The sequences of these four genes were identical in the cultivars ‘Gecheng’ and ‘Liuye.’ The four encoded proteins had highly conserved elements of TPSs, the DDxxD motif and the (N,D)D(L,I,V)x(S,T)xxxE motif, implying that they had the same capacity to bind the diphosphate group substrate (**Figure 5**). The N-terminal RR(x)_8_W motif, involved in producing cyclic monoterpenes and absent in TPSs that produce acyclic products, only appeared in OfTPS4 (**Figure 5**). Using the ‘ChloroP’ and ‘TargetP’ programs^4^, *Of*TPS1 and *Of*TPS3 were found to contain an extended N-terminus, recognized as a signal peptide, with a length of 47 and 38 amino acids, respectively. Phylogenetic analysis of the predicted amino acid sequences compared with TPS protein sequences in other species indicated that *Of*TPS1and *Of*TPS2 belong to the TPS-g subfamily, which lacks the RR(x)_8_W motif and mainly produce acyclic terpenes (**Figure 6**). *Of*TPS1 and *Of*TPS2 divided into two distinct clades. *Of*TPS1 grouped together with geraniol synthase and linalool synthase from *Olea europaea* and *Ocimum basilicam* in one clade, and *Of*TPS2 in the clade with linalool synthase, nerolidol/linalool synthase, myrcene synthase and ocimene synthase of *A. majus*. *Of*TPS3 and *Of*TPS4 fell into the TPS-b subfamily, covering angiosperm monoterpene synthases (**Figure 6**). *Of*TPS1, *Of*TPS3, and *Of*TPS4 showed highest amino acid sequence identity with geraniol synthase (90%), *Oe*TPS3 (90%), and *Oe*TPS2 (89%) from *O. europaea*, respectively. These results illustrate that they were the remaining uncharacterized TPSs and close to the similarly functional TPSs of *O. europaea*, also belonging to the Oleaceae family.

### Functional Identification of *OfTPS* Genes *in Planta*

**FIGURE 7:**
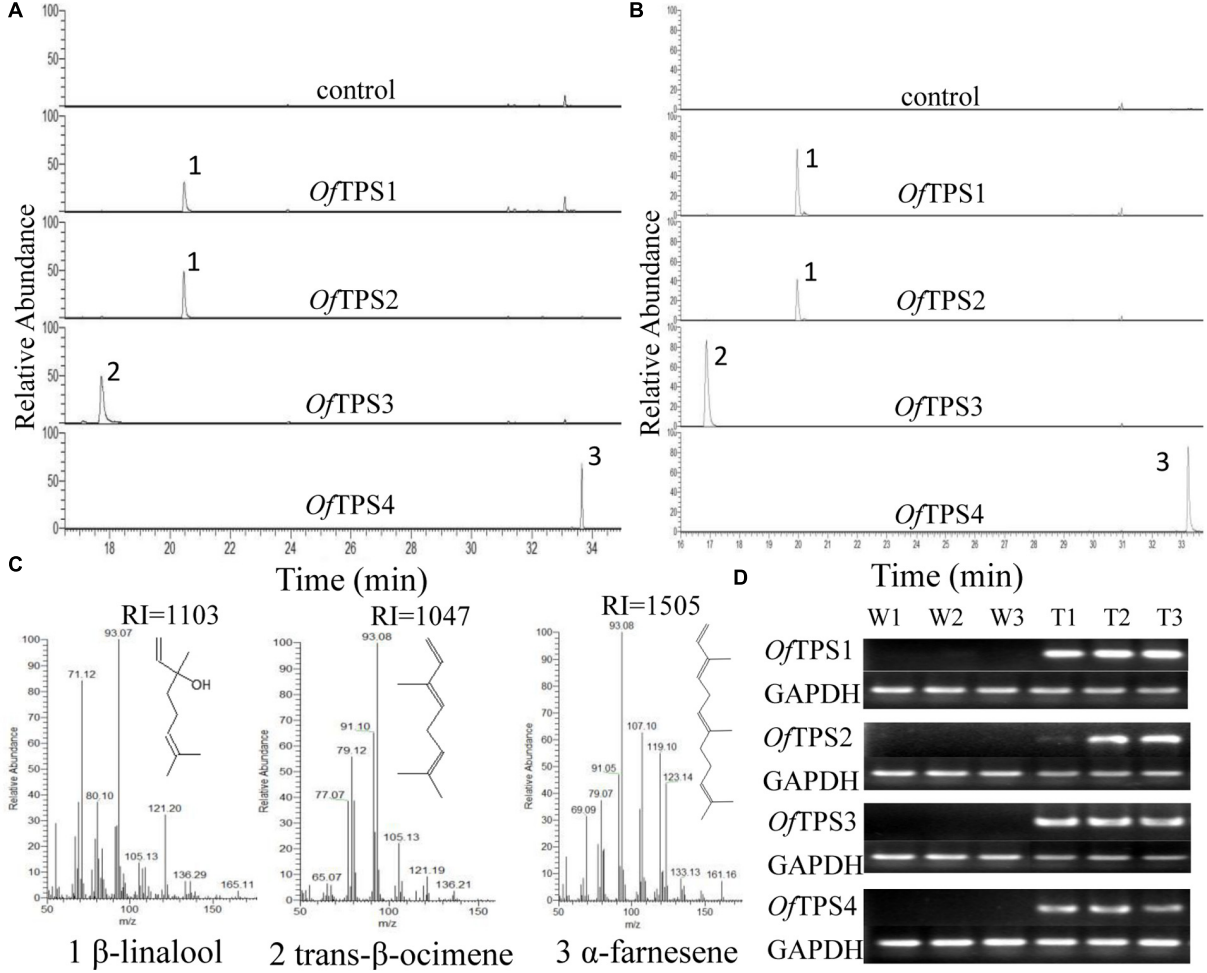
**Functional characterization of terpene synthases (TPSs) from *O. fragrans in planta.***(A)** GC trace of leaves of transiently expressed *N. benthamiana* plants. (B)** GC trace of leaves of transgenic *N. tabacum* plants. **(C)** Mass spectra of the products: (1) β-linalool, (2) *trans*-β-ocimene, (3) α-farnesene. Products were confirmed by comparison with authentic standards and/or GC-MS retention indices. **(D)** Semi-quantitative RT-PCR assay of *Of*TPS genes in transgenic *N. tabacum* plants.

To investigate the volatiles produced by *OfTPS*s *in planta*, transient plant expression (Hellens et al., 2005) was carried out by infiltration of *N. benthamiana* leaves using *A. tumefaciens* carrying pCAMBIA 2300::*OfTPS*s. Samples were taken 5–6 days after inoculation and emitted volatiles were analyzed by SPME headspace sampling. Over-expression of *OfTPS1* and *OfTPS2* produced large amounts of β-linalool, with no other products detected. Only *trans*-β-ocimene was detected in tobacco leaves over-expressing *OfTPS3*, and α-farnesene was the only product in those over-expressing *OfTPS4*. No related products were observed in control leaves infiltrated with empty binary vectors (**Figures 7A,C**).

Moreover, stably transformed *N. tabacum* plants were obtained through transformation with *A. tumefaciens* strain EHA105 harboring the pCAMBIA 2300::*OfTPS*s. Positive transgenic plants were further identified by semi-quantitative RT-PCR (**Figure 7D**). The volatile products of the *OfTPS* transformed plants were detected by SPME-GC-MS, and the results were the same as those from transient expression in plants (**Figure 7B**). These results suggest that both *OfTPS1* and *OfTPS2* are involved in synthesis of the same terpene product, β-linalool, and *OfTPS3* and *OfTPS4* are related to *trans*-β-ocimene and α-farnesene synthesis *in planta*, respectively.

## Discussion

Monoterpenes are common components of floral scent and play a role in attracting pollinators in plants (Dudareva and Pichersky, 2006). Previous studies focusing on floral volatiles composition from *O. fragrans* have identified about 20 monoterpenes and find that monoterpenes such as β-ocimene, linalool and linalool derivatives are the major components of floral volatiles in most cultivars (Cao et al., 2009; Sun et al., 2012; Xin et al., 2013). In an earlier study, we also identified these compounds as important aroma-active compounds in *O. fragrans* flowers, with the two cultivars of ‘Gecheng’ and ‘Liuye’ having distinct monoterpene emission profiles (Cai et al., 2014). The current study gives basically identical results: monoterpenes, especially *trans*-β-ocimene and linalool, make up a large amount of the floral volatiles in ‘Gecheng’ but only a trace in ‘Liuye’ (**Figure 1** and Supplemental Table S1). The results confirmed that ‘Liuye’ only produces small amounts of monoterpenes, unlike most *O. fragrans* cultivars, so this cultivar can be used as contrasting materials for our further study on the molecular mechanism of monoterpene biosynthesis.

### Monoterpenes Emission and Glycosylation in *O. fragrans* Flowers

The emission of floral volatiles in *A. majus* and the content of essential oil in *Lavandula* species can vary with flower development (Dudareva et al., 2000, 2003; Lane et al., 2010). We analyzed the emission and accumulation patterns of the dominant monoterpenes in two *O. fragrans* cultivars throughout the flowering stages. The highest levels of β-ocimene and linalool were found at the initial flowering stage in ‘Gecheng’ and at the full flowering stage in ‘Liuye,’ with emission of linalool derivatives reaching a peak at the initial or full flowering stage. These emission patterns were consistent with the other cultivars (Li et al., 2008) and the concentrated emission of monoterpenes coincided with the optimal time for organoleptic aroma and pollination in *O. fragrans* (Baldermann et al., 2010; Zhang, 2013). Moreover, the accumulation pattern of free linalool in the two cultivars was consistent with its emission, reaching the highest level in ‘Gecheng’ at the initial flowering stage and in ‘Liuye’ at the full flowering stage. Meanwhile, a parallel trend was observed between accumulation of free linalool oxides and their emission from the tight bud stage to the full flowering stage. These results suggested that both emission and accumulation of monoterpene are controlled by flower development, and the accumulation of free monoterpenes in flowers is directly proportional to their emission in *O. fragrans* flowers.

It has been reported that glycosylation might be involved in regulating the release of floral volatiles. For example, the decrease of glycosylated 2-phenylethanol in flowers correlating with rhythmic emission has demonstrated that glycosylation has an impact on floral 2-phenylethanol rhythmical release in *Rosa damascena* (Picone et al., 2004). But the glycosylation profile cannot account for the different rhythmic patterns of (E)-nerolidol accumulation and emission in *Actinidia chinensis* (Green et al., 2012). In this study, the accumulation and emission of linalool and its oxides in *O. fragrans* flowers shared parallel patterns from the tight bud stage to the full flowering stage. However, significant amounts of glycosylated linalool and its oxides were found during this period, and their accumulation continuously increased, especially at late full flowering stage when only a small amount of linalool and its oxides were released. These results showed that glycosylation was likely to have an impact on the linalool release in *O. fragrans* flowers at different flowering stages. Beside direct glycosylation, linalool tends to enzymatically convert to more stable linalool derivatives such as 8-hydroxylinalool and then is stored in plant tissues in glycosylated forms (Lewinsohn et al., 2001; Chen et al., 2010; Green et al., 2012). In *O. fragrans* flowers, a large amount of linalool derivatives, especially *cis*-8-hydroxylinalool and 8-hydroxylinalool, were found and depleted the pool of linalool. These results showed the complexity of linalool metabolism, and further research on the accumulation and emission of linalool and its derivative should consider the action of glucosidase, cytochrome, P450 or cyclase for further catalytic conversion.

### MEP Pathway Genes Analysis of *O. fragrans* Flowers

Monoterpene biosynthesis depends on the MEP pathway and the GPPS enzyme providing the GPP substrate (Chen et al., 2011). To discover the key gene controlling monoterpenes formation, the expression of genes involving in these enzymatic reactions were monitored in ‘Gecheng’ and ‘Liuye’ during flowering stages. It has been reported that, except for *DXS* and *IDI*, the other enzymes of the MEP pathway are encoded by single copy genes in *Arabidopsis* (Cordoba et al., 2009). Two distinctive forms *of CMK* and *IDS* have been found in gymnosperms such as *Ginkgo biloba* and *Pinus taeda* (Kim et al., 2008a,b). Two different gene expression patterns of *CMK, MCT*, and *IDS* in two cultivars throughout the flowering stages indicated that at least two forms exist in *O. fragrans*. This suggested that regulation of metabolic flux by a multigene is also present at the mid-point of the MEP pathway in addition to the first step of this pathway in *O. fragrans.*

As the over-expression of DXS in transgenic *A. thaliana* and tomato result in an increase in plastid isoprenoids such as diterpenes and carotenoids, the DXS enzyme has been considered as a rate-limiting enzyme for MEP pathway flux (Enfissi et al., 2005; Carretero-Paulet et al., 2006). In glandular trichomes of *Ocimum basilicum*, the transcript abundance of *DXS* correlates with oil yield which consists mostly of monoterpenes (Xie et al., 2008). In *V. vinifera*, *DXS* co-localizes with a major QTL for the accumulation of linalool, nerol and geraniol (Battilana et al., 2009). During the berry development, *DXS* expression shows a significant correlation with the accumulation profile of these monoterpenes (Battilana et al., 2011). However, in this study, *DXS* gene expression did not agree with the emission and accumulation profiles of monoterpenes from *O. fragrans* flowers. On the contrary, there was a higher expression of *DXS2* in ‘Liuye’ producing less monoterpenes from flowers. In the second step of the MEP pathway, DXR has also been suggested as a rate-limiting enzyme in *A. thaliana*, *Mentha piperita*, *O. basilicum* (Wildung and Croteau, 2005; Carretero-Paulet et al., 2006; Xie et al., 2008). However, the expression of *DXR* was also inconsistent with the monoterpenes production in *O. fragrans*. *HDS* and *IDS* is associated with foliar concentrations of the monoterpene 1,8-cineole in *Eucalyptus globulus* (Külheim et al., 2011). Here, there was no connection between gene expression of *HDS* and *IDS* and the production of monoterpenes. Therefore, we concluded that these MEP pathway genes controlling the pathway flux in other species was not the rate-limiting factor for monoterpene biosynthesis in *O. fragrans.* Unexpectedly, the level of expression of *CMK*2 and *MCT*2 was higher in ‘Gecheng’ than in ‘Liuye.’ There were no reports about the connection between CMK, MCT, and monoterpene production, however, their gene expression seemed to be consistent with monoterpene emission in *O. fragrans*, the roles of *CMK* and *MCT* need further analysis.

### *OfTPS* Genes Expression in *O. fragrans* Flowers

Transcriptional control of *TPS* genes has been shown to regulate the production of certain monoterpenes. For example, both spatial and temporal *AlstroTPS* expression correlates well with myrcene emission in scented *Alstroemeria* genotypes (Aros et al., 2012). Also, a concerted increase between expression of *LaLINS* and accumulation of linalool has been reported in the essential oil of *Lavandula angustifolia* during flower development (Lane et al., 2010). Similar results have been found in plants such as *A. majus* (Dudareva et al., 2003) and *Origanum vulgare* (Crocoll et al., 2010). In this study, *TPS2* and *TPS4* expression showed a drastic increase from full flowering stage to late full flowering stage but no difference between ‘Gecheng’ and ‘Liuye’ during flowering stages, which was not consistent with the emission and accumulation of monoterpene in the two cultivars. Interestingly, expression of *TPS1* and *TPS3* was more than 100 fold higher in ‘Gecheng’ as compared to ‘Liuye,’ and their expression was in agreement with the production of monoterpene during flowering. These results indicated a direct contribution of *TPS1* and *TPS3* to monoterpenes production in flowers of *O. fragrans.* Carotenoid and monoterpene biosynthesis share the same pathway, which indicates a possible competition for the IPP and DMAPP precursors (Botella-Pavía et al., 2004; Dudareva et al., 2013). However, although the production of monoterpenes in ‘Liuye,’ containing trace amounts of carotenoids, was low and ‘Gecheng,’ with large mounts of carotenoids still produced a lot of monoterpenes, this was only related to *TPS* genes expression. The results further demonstrated that the transcript levels of *TPS* genes on monoterpene production had a greater effect compared with the competition of carotenoid synthesis. This led to the conclusion that transcriptional regulation of *TPS* genes, especially *TPS1* and *TPS3*, was likely to be the most important mechanism for controlling production of the monoterpenes linalool or β-ocimene in *O. fragrans* flowers.

### Sequence and Functional Characterization of *O. fragrans TPS* Genes

The putative functions of the four isolated TPSs from *O. fragrans* were initially predicted according to the conserved motifs and amino acid sequence similarity with known TPSs from other species. The four *Of*TPSs shared the conserved motifs DDxxD and (N,D)Dxx(S,T)xxxE) with other TPSs. *Of*TPS1 and *Of*TPS3 had the typical putative plastid targeting signal peptides but lacked the RR(x)_8_W motif in the N-terminal, which indicated they probably encoded acyclic monoterpene synthases. Phylogenetic analysis placed *Of*TPS1 in the TPS-g subfamily defined by monoterpene synthases, that produce acyclic scent compounds such as myrcene and ocimene in *A. majus* (Dudareva et al., 2003), and linalool in *A. thaliana* (Chen et al., 2003) and *V. vinifera* (Martin et al., 2010). Although *Of*TPS2 also belonged to the TPS-g subfamily, it was not clear whether this gene encodes a mono-TPS or sesqui-TPS, due to its unpredictable signal peptide and clustering with the bifunctional linalool/nerolidol synthase of *A. majus* (Nagegowda et al., 2008), *V. vinifera* (Martin et al., 2010), and *A. chinensis* (Green et al., 2012). *Of*TPS3 and *Of*TPS4 clustered into the TPS-b subfamily in which all characterized TPSs are either monoterpene synthases, including all *A. thaliana* monoterpene synthases except linalool synthase (Chen et al., 2003) and *V. vinifera* ocimene synthases (Martin et al., 2010), or isoprene synthases (Chen et al., 2011). But *Of*TPS4 clustered with α-farnesene synthase from *Malus domestica* (Nieuwenhuizen et al., 2013) and *V. vinifera* (Martin et al., 2010). Of the four *Of*TPS proteins, only *Of*TPS4 had the RR(x)_8_W motif that is present in all cyclic monoterpene synthases and most sesquiterpene and diterpene synthases (Hyatt et al., 2007), which may play a role in triggering isomerization-cyclization or act to stabilize the protein (Whittington et al., 2002a,b). Moreover, the unapparent signal peptide and high homology with α-farnesene synthase in phylogenetic analysis suggested that *Of*TPS4 was most likely a sesquiterpene synthase.

Transient and stable expression in tobacco leaves further confirm the function of *OfTPS* genes *in planta*, which could partly explain the production of major monoterpene volatiles in *O. fragrans* flowers. Over-expression of *OfTPS1* and *OfTPS3* genes in tobacco leaves resulted in the formation of only β-linalool and *trans*-β-ocimene, respectively. *Of*TPS2, that may be a bifunctional linalool/nerolidol synthase, produced β-linalool exclusively in over-expressing tobacco leaves. As previously speculated, sesquiterpene α-farnesene was the sole product emitted from tobacco leaves over-expressing *OfTPS4*. Most of the TPS enzymes from *V. vinifera* are multiple-product enzymes that can act on one substrate to produce more than one product (Martin et al., 2010). However, there was only a single product of the four *OfTPS*s in the over-expressing tobacco leaves. The TPS enzyme, that produces the same single product, has also been found in *V. vinifera* and *M. domestica* (Pechous and Whitaker, 2004; Martin et al., 2010; Nieuwenhuizen et al., 2013). *Of*TPS1, *Of*TPS3, and *Of*TPS4 showed high homology with *Oe*GES, *Oe*TPS3, and *Oe*TPS2 from *O. europaea*, respectively (**Figure 6**). The homologous TPS proteins from *O. fragrans* and *O. europaea* had entirely different enzyme activities. Heterologous expression in a bacterial system has demonstrated that OeGES is a geraniol synthase only producing geraniol, and OeTPS2 and OeTPS3 have no enzymatic activity (Vezzaro et al., 2012). Fischer et al. (2013), expressing *O. basilicum* geraniol synthase in *V. vinifera*, *A. thaliana*, *N. benthamiana* and a microbial system, has shown that the genetic background of the heterologous expression host could have an influence on minor products but not on the main product. Nevertheless the major monoterpene compounds (β-linalool and *trans*-β-ocimene) found in *O. fragrans* flowers have been detected in tobacco leaves over-expressing *OfTPS1*, *OfTPS2*, and *OfTPS3*. Moreover, gene expression of *OfTPS1* and *OfTPS3* are closely correlated to the accumulation or emission of β-linalool and *trans*-β-ocimene in *O. fragrans* flowers. Therefore, we conclude that *OfTPS1* and *OfTPS3* are separately the key genes associating with β-linalool and *trans*-β-ocimene production in *O. fragrans* flowers.

This study provided the molecular basis for the production of major monoterpenes in *O. fragrans.* The monoterpenes emission and accumulation profiles open the way to further understand the role of monoterpenes in the physiology and pollination biology of *O. fragrans* flowers. Understanding the expression of the MEP pathway genes in relation to monoterpene metabolism could generate vital information regarding the regulation of monoterpene biosynthesis in higher plants. The findings on the key TPS genes responsible for formation of the major monoterpenes would be a great advantage for breeding and manipulation of scent related-enzymes to enhance the ornamental and economic value in *O. fragrans* as well as in other plants in the future.

## Author Contributions

XZ, CW designed the research; XZ, CL, XC, and JZ performed the research; XZ, RZ, and CL analyzed the data; XZ wrote the article; CW, JL, and RZ critically read the article.

## Conflict of Interest Statement

The authors declare that the research was conducted in the absence of any commercial or financial relationships that could be construed as a potential conflict of interest.

## Footnotes

1 http://www.ncbi.nlm.nih.gov/gorf/orfig.cgi

2 http://www.cbs.dtu.dk/services/ChloroP/

3 http://www.cbs.dtu.dk/services/TargetP/

4 http://www.cbs.dtu.dk/services/
