# Supplementary material for: Emission and Accumulation of Monoterpene and the Key Terpene Synthase (TPS) Associated with Monoterpene Biosynthesis in Osmanthus fragrans Lour
Source: Front Plant Sci. 2016 Jan 12;6:1232. doi: 10.3389/fpls.2015.01232 (PMC4709469; doi:10.3389/fpls.2015.01232)
Supplement: Supplementary file 1 [file Data_Sheet_1.DOCX]

**Supplemental TABLE 1 | Volatile compounds from flowers of ‘Liuye’ and ‘Gecheng’ cultivars of *O. fragrans***

|  | **Compound** | **Formula** | **CAS** | **LRI** | **LRI^a^** | **Relative Content (%) ±SE** | |
| --- | --- | --- | --- | --- | --- | --- | --- |
|  |  |  |  |  |  | **Liuye** | **Gecheng** |
| 1 | 6-Methyl-5-heptene-2-one | C_8_H_14_O | 110-93-0 | 981 | 987 | 0.20±0.08 | - |
| **2** | **β-Myrcene** | **C_10_H_16_** | **123-35-3** | **988** | **991** | **0.04±0.03** | **0.5±0.11** |
| 3 | (E)-3-Hexen-1-ol, acetate | C_8_H_14_O_2_ | 3681-82-1 | 1005 | 1005 | 0.25±0.01 | 0.08±0.01 |
| 4 | 1-Methyl-3-(1-methylethyl)-benzene | C_10_H_14_ | 535-77-3 | 1018 | 1022 | 0.05±0.00 | - |
| **5** | **D-Limonene** | **C_10_H_16_** | **5989-27-5** | **1027** | **1035** | **0.02±0.01** | **0.15±0.01** |
| **6** | **cis-β-Ocimene** | **C_10_H_16_** | **3338-55-4** | **1037** | **1038** | **0.25±0.08** | **1.00±0.38** |
| **7** | **trans-β-Ocimene** | **C_10_H_16_** | **3779-61-1** | **1047** | **1048** | **1.44±0.55** | **44.37±3.07** |
| **8** | **γ-Terpinen** | **C_10_H_16_** | **99-85-4** | **1057** | **1061** | **0.02±0.00** | **0.43±0.18** |
| **9** | **cis-Linalool oxide (furan)** | **C_10_H_18_O_2_** | **5989-33-3** | **1071** | **1074** | **0.20±0.07** | **2.64±1.36** |
| **10** | **Terpinolene** | **C_10_H_16_** | **586-62-9** | **1083** | **1086** | **-** | **0.08±0.03** |
| **11** | **trans-Linalool oxide (furan)** | **C_10_H_18_O_2_** | **34995-77-2** | **1090** | **1088** | **0.17±0.04** | **3.17±1.50** |
| 12 | (Z)-Butanoic acid, 2-pentenyl ester | C_9_H_16_O_2_ | 42125-13-3 | 1098 | 1089 | 0.14±0.08 | - |
| **13** | **β-Linalool** | **C_10_H_18_O** | **78-70-6** | **1103** | **1102** | **2.27±1.14** | **23.21±14.88** |
| **14** | **Hotrienol** | **C_10_H_16_O** | **29957-43-5** | **1106** | **1108** | **0.27±0.11** | **-** |
| **15** | **6-Ethenyldihydro-2,2,6-trimethyl-2H-pyran-3(4H)-one** | **C_10_H_16_O_2_** | **33933-72-1** | **1110** | **1107** | **-** | **0.30±0.12** |
| **16** | **1,3,8-p-Menthatriene** | **C_10_H_14_** | **21195-59-5** | **1115** | **1111** | **0.03±0.03** | **0.13±0.07** |
| **17** | **Cosmene** | **C_10_H_14_** | **460-01-5** | **1138** | **1134** | **0.31±0.11** | **2.03±0.80** |
| **18** | **Allo-ocimene** | **C_10_H_16_** | **673-84-7** | **1146** | **1134** | **0.16±0.05** | **1.78±0.59** |
| 19 | cis-3-Hexenyl iso-butyrate | C_10_H_18_O_2_ | 41519-23-7 | 1150 | 1145 | 0.37±0.28 | - |
| **20** | **cis-Linalol oxide (pyran)** | **C_10_H_18_O_2_** | **14009-71-3** | **1172** | **1174** | **0.07±0.00** | **0.26±0.12** |
| **21** | **trans-Linalool oxide (pyran)** | **C_10_H_18_O_2_** | **14049-11-7** | **1178** | **1173** | **0.05±0.00** | **0.71±0.35** |
| 22 | (Z)-Butanoic acid, 3-hexenyl ester | C_10_H_18_O_2_ | 16491-36-4 | 1191 | 1186 | 0.67±0.26 | - |
| 23 | Butanoic acid, hexyl ester | C_10_H_20_O_2_ | 2639-63-6 | 1196 | 1191 | 0.08±0.02 | - |
| 24 | n-Valeric acid cis-3-hexenyl ester | C_11_H_20_O_2_ | 35852-46-1 | 1235 | 1236 | 0.15±0.09 | - |
| 25 | cis-3-Hexenyl isovalerate | C_11_H_20_O_2_ | 35154-45-1 | 1242 | 1240 | 0.06±0.00 | - |
| 26 | 2,3-Dihydro-2-methylbenzofuran | C_9_H_10_O | 1746-11-8 | 1289 | 1306 | 0.11±0.03 | 0.09±0.00 |
| 27 | Edulan I | C_13_H_20_O | 41678-29-9 | 1330 | 1315 | 0.10±0.04 | - |
| 28 | 1,2,3,4-tetrahydro-1,4,6-  trimethylnaphthalene | C_13_H_18_ | 22824-32-4 | 1383 | - | 0.37±0.10 | 0.07±0.02 |
| 29 | α-Ionone | C_13_H_20_O | 127-41-3 | 1430 | 1428 | 9.39±2.81 | 1.93±0.63 |
| 30 | Dihydro-β-Ionone, | C_13_H_22_O | 17283-81-7 | 1442 | 1443 | 2.96±0.71 | 1.15±0.57 |
| 31 | γ-Decalactone | C_10_H_18_O_2_ | 706-14-9 | 1472 | 1467 | 6.12±4.34 | 3.20±1.11 |
| 32 | trans-β-Ionone | C_13_H_20_O | 79-77-6 | 1490 | 1487 | 68.30±10.48 | 7.99±1.81 |
| 33 | β-Ionone | C_13_H_20_O | 14901-07-6 | 1494 | 1489 | 0.17±0.03 | - |
|  | **Total compounds identified in both cultivars** | | | | | **33** | **33** |
|  | **Kinds of compounds identified in each cultivar** | | | | | **31** | **22** |
|  | **Relative content of identified compounds (%)** | | | | | **94.67±2.29** | **83.63±7.71** |
|  | **Total monoterpenes in both cultivars** | | | | | **17** | **17** |
|  | **Kinds of monoterpenes in each cultivar** | | | | | **14** | **16** |
|  | **Kinds of monoterpenes in both cultivars** | | | | | **13** | **13** |
|  | **Relative content of monoterpenes (%)** | | | | | **5.03±2.79** | **69.34±6.0** |
| Dash indicates not detected; monoterpene compounds in bold; LRI^a^ (linear retention index) from published literature. | | | | | | | |

**Supplemental TABLE 2 | Gene-specific terpene synthase primers used for cloning and expression analyses**

| Primer | Sequence |
| --- | --- |
| For 3' RACE PCR |  |
| *OfTPS1*-3-1  *OfTPS1*-3-2 | 5'-AGCCTTATGCTTCCGCTTGCTCCGC-3'  5'-ACAGTCGATGCCTCAGTTGGCGCCGC-3' |
| *OfTPS2*-3-1  *OfTPS2*-3-2 | 5'-TGCTTCAGGGCACTTGCCAACCTCCAAT-3'  5'-GCTCGTCCACCTCTTCTTTCTTTTGGGG-3' |
| *OfTPS3*-3-1  *OfTPS3*-3-2  *OfTPS3*-3-3 | 5'-ACGAAGATGGGGAATACATTGTGCTGCC-3'  5'-AATGGGTGCACGAGTGAGACATGCC-3'  5'-CCATTTCAGCGCAGAATGCAAAGGC-3' |
| *OfTPS4*-3-1  *OfTPS4*-3-2 | 5'-CTGGTTTGTCCGTTGCATTTGAGTGTCG-3'  5'-CATGGCTCAAGCCCAACAGCAAAAGG-3' |
| For 5' RACE PCR |  |
| *OfTPS1*-5-1  *OfTPS1*-5-2 | 5'-GGCAGTTCCTAGATCATCCCAGAGACGG-3'  5'-GGGCTGGATCGTGTTCACTTTCTGTAG -3' |
| *OfTPS2*-5-1  *OfTPS2*-5-2 | 5'-CTTCTGAGGAGTAGCTTTGCACGCAACA-3'  5'-GCAGCCACCGAGGATATGAGTTCTG-3' |
| *OfTPS3*-5-1  *OfTPS3*-5-2  *OfTPS3*-5-3 | 5'-ACGAAGATGGGGAATACATTGTGCTGCC-3'  5'-AATGGGTGCACGAGTGAGACATGCC-3'  5'-CCATTTCAGCGCAGAATGCAAAGGC-3' |
| *OfTPS4*-5-1  *OfTPS4*-5-2  *OfTPS4*-5-3  *OfTPS4*-5-4 | 5'-CCCTTTTCCTGTTGAATTTGCAGATCCA-3'  5'-GCTGCTGAATTTCTGTGTGATTCCACC-3'  5'-TCTCCTTGAGATCCTTTTGCTGTTGGGC-3'  5'-GTGCAGCGTGGTAGAGGTTTTCTTCC -3' |
| For ORF full length |  |
| *OfTPS1*-LF  *OfTPS1*-LR | 5'-CTCCTCTCCATTACACTAACGCAATATGGAGTG-3'  5'-CCAATCGAATTACACAAGGCCTAAGGATACATG-3' |
| *OfTPS2*-LF  *OfTPS2*-LR | 5'-TACATTTCTATCTGACATGGCAGTC-3'  5'-TTTATGGACGGTGCACCAAGAAAGTT-3' |
| *OfTPS3*-LF  *OfTPS3*-LR | 5'-ACATGGGGATGGGCATAATAAACATAG-3'  5'-TTTTAGATAGCCCCAATAACAGAACACC-3' |
| *OfTPS4*-LF  *OfTPS4*-LR | 5'-CATGGGGATCTTCAAAACTACTTATCTC-3'  5'-TGAACTGATGCTCGTTATTAACAAAGC-3' |
| For real-time qRT-PCR |  |
| *OfDXS*-1-F0  *OfDXS*-1-R0 | 5'-CTATCTCAGCAGGACTAGGAATGGC-3'  5'-GGGTAACGAGACTTGTTTGTTGTCA-3' |
| *OfDXS*-2-F0  *OfDXS*-2-R0 | 5'-ACAGACCAAGCTGCTTTAGGTTTCC-3'  5'-CGGGAATACGTTATGGGATTTTAGC-3' |
| *OfDXR*-F0  *OfDXR*-R0 | 5'-CCATGACAGGGGTTCTTAGCG-3'  5'-CGTAATCCCGAGCCCACAAGT-3' |
| *OfMCT*-1-F0  *OfMCT*-1-R0 | 5'-ACTATCAAAGAGGCAAATGGTGAAT-3'  5'-CACAGGATGTTTAAGGTGCTCTATG-3' |
| *OfMCT*-2-F0  *OfMCT*-2-R0 | 5'-CATCGTCGGTATTCATCAAAGAAGC-3'  5'-ACCAAGTAACTCGCACAAATTAGCC-3' |
| *OfCMK*-1-F0  *OfCMK*-1-R0 | 5'-GGGTTCGATTTTCAACAATCAGAAC-3'  5'-ATCCACTTCATCGGCTAGTTTATTG-3' |
| *OfCMK*-2-F0  *OfCMK*-2-R0 | 5'-TGGTGGTGGTAGTAGTAATGCCG-3'  5'-TGTACAGTAGGCTGCTCCATGAGAG-3' |
| *OfMECPS*-F0  *OfMECPS*-R0 | 5'-CCCGACAATGACCCTAAATGGA-3'  5'-TCTGCACCAAGTAGCTCGCACA-3' |
| *OfHDS*-F0  *OfHDS*-R0 | 5'-GCAGTTGACGAAGCCATTACCC-3'  5'-TCGCCACGCACAGAAACCAC-3' |
| *OfIDS*-1-F0  *OfIDS*-1-R0 | 5'-TTTTGCAGGAAAGTACATCATTGTG-3'  5'-ACCAAGTTTTACAAGATCCGTGTTT-3' |
| *OfIDS*-2-F0  *OfIDS*-2-R0 | 5'-GAGGCAACGTATGTGTGCGATTA-3'  5'-AAACCCCTCGGATACAGCATATTTA-3' |
| *OfIDS*-3-F0  *OfIDS*-3-R0 | 5'-TCGTCGGCTACTCTGGAGTCG-3'  5'-CTTGTGTACTCGCGGTTCATGAG-3' |
| *OfIDI*-F0  *OfIDI*-R0 | 5'-CAGATGCTGGTGAGGGTGGTATTA-3'  5'-TGCTTTGCTCAGAGTCCCTTTCT-3' |
| *OfGPPS*-F0  *OfGPPS*-R0 | 5'-GCTCATGAACACCTGCCAATCA-3'  5'-GGAACCATACCATCTCCCGTAAGA-3' |
| *OfTPS1*-F0  *OfTPS1*-R0 | 5'-GAATATGCTACAGAAAGTGAACACGA-3'  5'-GAAGTCCCACAGTCCATAAAAAGC-3' |
| *OfTPS2*-F0  *OfTPS2*-R0 | 5'-CTATTCTCCGTCTCTGGGATGACTT-3'  5'-TTCACGTAGCACTCTATGTACGAACC-3' |
| *OfTPS3*-F0  *OfTPS3*-R0 | 5'-CTCTACTTCACCCTCCTTAGACAACA-3'  5'-TATTCTCTCCATTATATGCAACACGT-3' |
| *OfTPS4*-F0  *OfTPS4*-R0 | 5'-GGTTTGTCCGTTGCATTTGAGTG-3'  5'-AGATCCTTTTGCTGTTGGGCTTG-3' |
| *OfActin*-F0  *OfActin*-R0 | 5'-ATTAGTCCTCTTCCAGCCTTCTTTG-3'  5'-ATTATTTCCTTGCTCATACGGTCAG-3' |
